# Effect of ribbon width on electrical transport properties of graphene nanoribbons

**DOI:** 10.1186/s40580-018-0139-0

**Published:** 2018-03-15

**Authors:** Kyuhyun Bang, Sang-Soo Chee, Kangmi Kim, Myungwoo Son, Hanbyeol Jang, Byoung Hun Lee, Kwang Hyeon Baik, Jae-Min Myoung, Moon-Ho Ham

**Affiliations:** 1 Department of Materials Science and Engineering, Yonsei University, Seoul, 03722 Republic of Korea; 2 School of Materials Science and Engineering, Gwangju Institute of Science and Technology, Gwangju, 61005 Republic of Korea; 3 School of Materials Science and Engineering, Hongik University, Sejong, 30016 Republic of Korea

**Keywords:** Graphene, Graphene nanoribbon, Si nanowire, Electrical transport

## Abstract

There has been growing interest in developing nanoelectronic devices based on graphene because of its superior electrical properties. In particular, patterning graphene into a nanoribbon can open a bandgap that can be tuned by changing the ribbon width, imparting semiconducting properties. In this study, we report the effect of ribbon width on electrical transport properties of graphene nanoribbons (GNRs). Monolayer graphene sheets and Si nanowires (NWs) were prepared by chemical vapor deposition and a combination of nanosphere lithography and metal-assisted electroless etching from a Si wafer, respectively. Back-gated GNR field-effect transistors were fabricated on a heavily p-doped Si substrate coated with a 300 nm-thick SiO_2_ layer, by O_2_ reactive ion etching of graphene sheets using etch masks based on Si NWs aligned on the graphene between the two electrodes by a dielectrophoresis method. This resulted in GNRs with various widths in a highly controllable manner, where the on/off current ratio was inversely proportional to ribbon width. The field-effect mobility decreased with decreasing GNR widths due to carrier scattering at the GNR edges. These results demonstrate the formation of a bandgap in GNRs due to enhanced carrier confinement in the transverse direction and edge effects when the GNR width is reduced.

## Introduction

Over the past decade, graphene has emerged as a promising candidate for application in future nanoelectronics due to its excellent material properties such as high carrier mobility, excellent mechanical flexibility, high thermal conductivity, and high optical transparency [1–7]. In particular, graphene is regarded as an outstanding channel material because of its electron mobility as high as 200,000 cm^2^ V^−1^ s^−1^ [8]. In spite of the superior properties of graphene, there are several challenges to be overcome for practical applications in electronic devices.

As graphene is intrinsically a semimetal with zero bandgap, the formation of a bandgap is necessary to achieve a sufficiently high on/off current ratio when used as a channel in a field-effect transistor (FET). Several routes to bandgap tuning in graphene have been reported, such as chemical doping of graphene, application of a vertical electric field in bilayer graphene, and patterning graphene into a narrow ribbon structure [9–11]. Among these, patterning graphene into nanoribbons can open a bandgap that is tunable by narrowing the ribbon width to less than 50 nm. Graphene nanoribbons (GNRs), which are narrow strips of graphene, exhibit semiconducting properties due to the quantum confinement and edge effects. Han et al. experimentally demonstrated that the energy bandgap of GNRs scale inversely with the channel width [12]. Wang et al. also reported a high on/off current ratio of up to ~ 10^4^ in GNR FETs with sub-5 nm widths at room temperature [13]. The fabrication methods for GNRs include e-beam lithography [12–15], block copolymer lithography [16], and use of a nanowire (NW) etch mask [17–19] on graphene sheets, and also through unzipping of carbon nanotubes [20–24]. In this study, we investigated the ribbon-width dependence of electrical transport properties of GNRs. Si NWs, fabricated by a combination of polystyrene (PS) nanosphere lithography and metal-assisted electroless etching, were used as etch masks to fabricate a nanoribbon structure by exposing graphene to oxygen plasma. The Si NWs were aligned on the graphene between the two electrodes by electric-field-assisted assembly, and oxygen plasma was used to transfer NW morphology onto the graphene by removing unprotected graphene. The ribbon widths of the GNRs were controlled by the diameters of the Si NWs, and the electrical transport properties of these GNRs with different ribbon widths were investigated.

## Experimental

### Synthesis and transfer of monolayer graphene

Cu foil (Alfa Aesar) was used as a substrate for graphene growth. The Cu foil was annealed at 1000 °C for 30 min under 10 sccm of H_2_ gas flow to increase the Cu grain size and ensure the removal of native oxide and a smooth Cu surface. Graphene was grown on Cu foil at 1000 °C using a mixture of 20 sccm of CH_4_ and 50 sccm of H_2_ by low-pressure chemical vapor deposition (CVD) [16, 25]. To prepare for the graphene transfer to a Si/SiO_2_ substrate, the surface of the graphene on Cu was coated with poly(methyl methacrylate) (PMMA, MicroChem) as a transfer medium. The PMMA-coated samples were baked at 60 °C for 5 min. The Cu was wet-etched using a copper etchant (CE-100, Transene Co., Inc.), resulting in graphene/PMMA films floating on the etchant. These films were then collected onto Si/SiO_2_ substrates, and the PMMA layers were removed with acetone, yielding Si/SiO_2_/graphene.

### Fabrication of Si NWs with different diameters

Si NWs were prepared by nanosphere lithography, which is a technique for generating hexagonally close-packed nanoscale patterns of nanometer-sized PS spheres on a Si wafer. PS beads with different diameters of 100, 300, and 460 nm were dispersed in methanol with Triton X-100 as a surfactant. To make the surface of the Si substrate hydrophilic, the substrate was immersed in a piranha solution (1:3 of 30% H_2_O_2_:H_2_SO_4_) at 100 °C. Then, the substrate was rinsed with deionized water and spin-coated with the PS bead suspension at 3000 rpm to form a monolayer of PS beads on the Si substrate. The large-area ordered and close-packed monolayer assembly of PS beads on the Si substrate was transformed into a non-close-packed arrangement by reducing the diameter of the PS spheres using O_2_ reactive ion etching (RIE). The e-beam evaporator was used to deposit 40 nm-thick Ag films as catalysts to form Si NWs from the substrate, and the substrate was subsequently sonicated in chloroform for 20 min to remove the PS spheres. Wet etching was performed at 50 °C in a solution of 4.5 M HF and 0.5 M H_2_O_2_ for 20–30 min, yielding vertically aligned Si NW arrays. Finally, the Ag films were removed by immersion in boiling aqua regia (3:1 of HCl:HNO_3_) at 100 °C for 20 min.

### Fabrication of GNR FETs

A large-area monolayer graphene on a heavily p-doped Si substrate (bottom gate) coated with a 300 nm-thick SiO_2_ layer (gate dielectric) was used as a starting material to fabricate GNR FETs. First, the source/drain pads were patterned by photolithography, followed by deposition of Ti/Au as the electrode metal on the graphene by e-beam evaporation. The channel length of the FETs was 8 μm. The Si NWs were raked out from the vertically aligned Si NW arrays prepared on the Si substrate and dissolved in isopropyl alcohol (IPA). This Si NW suspension was sonicated for 10 min and centrifuged at 2500 rpm for 10 min. Then, the Si NWs were aligned on graphene between the two electrodes by a dielectrophoresis method. The assembly of Si NWs was performed by dispensing a dilute suspension of the Si NWs onto the substrates with applied voltages of ± 10 V at frequencies of 1–100 kHz (81110A, Agilent Technologies). O_2_ plasma etching was used to selectively etch an unprotected graphene region away and leave a ribbon structure of graphene underneath the Si NW mask, leading to the formation of GNRs. After that, the NW mask was removed by sonication in IPA, resulting in GNR FETs.

### Characterization

The monolayer graphene was characterized by Raman spectroscopy (LabRAM HR Evolution, Horiba Jovin-Yvon) using a laser with an excitation wavelength of 532 nm. The morphological properties of graphene, Si NWs, and GNRs were investigated by optical microscopy (BX51, Olympus), field-emission scanning electron microscopy (FESEM, JSM-7500F, JEOL), and atomic force microscopy (AFM, XE-100, Park Systems). The electrical characteristics of the GNR FETs were studied with a semiconductor parameter analyzer (E5270B, Agilent Technologies).

## Results and discussion

**Fig. 1:**
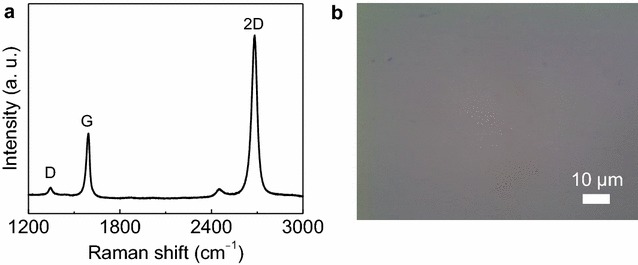
**a** Raman spectrum and **b** optical image of a monolayer graphene sheet transferred on a Si/SiO_2_ substrate

Raman spectroscopy was performed to confirm the formation of graphene and to obtain information about the quality and the number of graphene layers. Figure 1a shows a typical Raman spectrum of monolayer graphene. A symmetric 2D band centered at ~ 2680 cm^−1^ with a full width at half maximum of ~ 33 cm^−1^ was observed. The peak intensity ratio of the 2D and G bands was equal to 2, indicating high-quality monolayer graphene. The weak peak located at 1345 cm^−1^ corresponded to the D band of graphitic carbon species, the intensity of which was associated with the level of defects in the crystalline structure of the graphene layers. This weak D band confirmed the high quality of the synthesized graphene films. Figure 1b shows an optical image of the uniform graphene sheet transferred on a SiO_2_ layer.

**Fig. 2:**
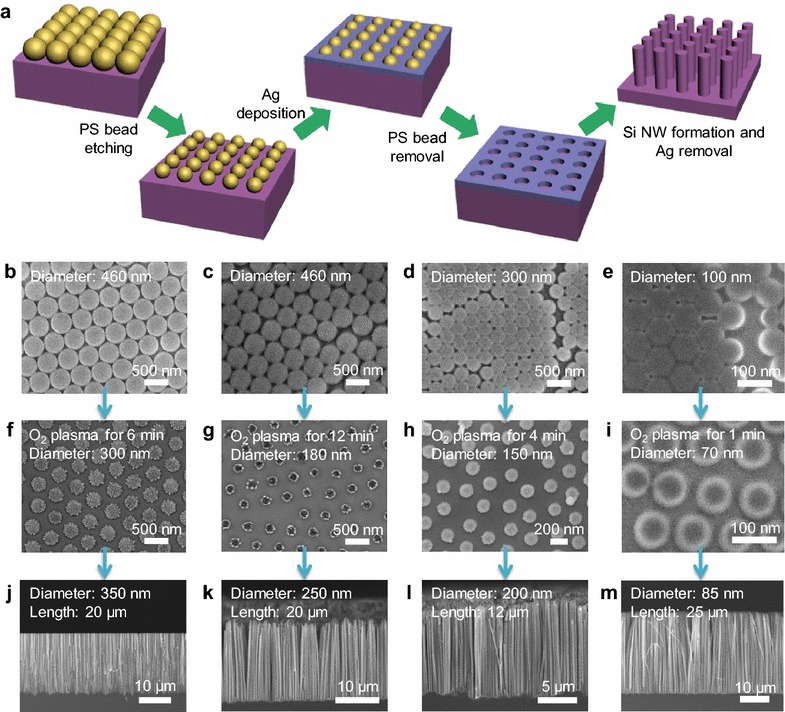
**a** Schematic illustration of the preparation of a Si NW array using a Ag film with uniform-sized holes as an etch mask, where PS beads were used for patterning the Ag film. **b**–**m** FESEM images of the process steps for fabricating vertically aligned, ordered Si NW arrays

**Fig. 3:**
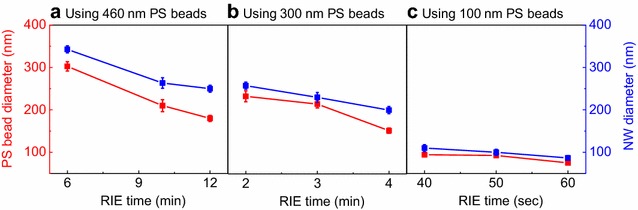
Variations of diameter of Si NWs formed with PS beads with diameters of **a** 460, **b** 300, and **c** 100 nm as a function of etching time

The fabrication of ordered Si NW arrays using nanosphere lithography involves several distinct steps, which are schematically described in Fig. 2a. Figure 2b–m show SEM images of each step in Si NW fabrication. PS beads, which assemble in close-packed hexagonal patterns, were used as masks for patterning Ag catalysts (Fig. 2b–d). The diameter of the PS spheres can be reduced to the desired size by varying the etching duration time of the O_2_ RIE process, which results in the formation of PS bead arrays that are no longer close-packed (Fig. 2f–i). When PS beads with a diameter of 460 nm were etched for 6 and 12 min, PS bead arrays with diameters of 300 and 180 nm, respectively, were obtained. For PS beads with a starting diameter of 300 nm, the diameter was reduced to 150 nm after etching for 4 min. The oxygen plasma etching of 100 nm PS beads for 60 s reduced the diameter to 70 nm. Then, Ag film as a catalyst was coated on the Si substrate with the PS bead assembly [26, 27], and the PS spheres were etched away from the substrate, yielding the Ag film with a hexagonal array of holes. The dimensions of the holes were well matched with the diameters of the size-reduced PS beads. After the wet etching process, vertically aligned, ordered Si NW arrays were produced due to the structure of the catalytic Ag mesh (Fig. 2j–m). The diameter of the Si NWs was mainly determined by the size of the nanohole arrays on the Ag films, which could be controlled by changing the size and etching time of the PS beads. Figure 3 shows the diameter variations of the fabricated Si NWs as a function of O_2_ plasma etching time for the samples using PS beads with diameters of 460, 300, and 100 nm. The diameters of the PS beads and the resulting Si NWs approximately linearly decreased with increasing O_2_ plasma etching times.

**Fig. 4:**
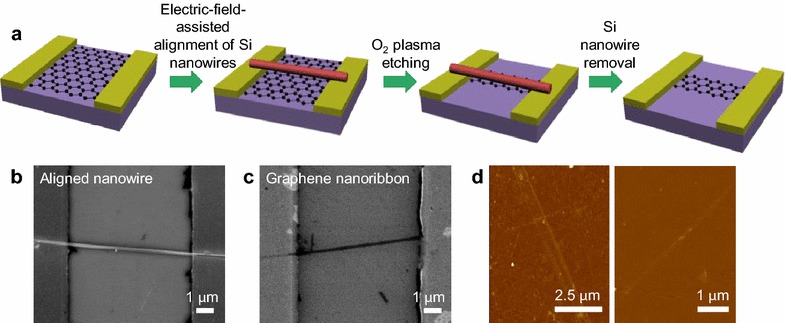
**a** Schematic illustration of the fabrication of a GNR FET using a Si NW as a hard etch mask. FESEM images of **b** a Si NW aligned on graphene between two electrodes, where unscreened graphene was removed, and **c** a GNR after the removal of the Si NW. **d** AFM images of GNRs with two different ribbon widths (left: 500 nm and right: 100 nm)

Figure 4a schematically shows a process sequence for GNR FET fabrication. Back-gated FETs were fabricated from the graphene sheets, which were synthesized on Cu foil by CVD and transferred onto a Si/SiO_2_ substrate by a PMMA-assisted wet-transfer method [16, 25], using standard photolithography techniques. Then, the Si NWs were aligned on graphene between the two electrodes by a dielectrophoresis method [28, 29], and O_2_ plasma etching was carried out to form GNRs by selectively removing unprotected graphene. Figure 4b shows an SEM image of a fabricated GNR having a Si NW, where unscreened graphene was etched away underneath the Si NW. After removing the Si NW from the substrate by sonication, the FETs were fabricated with single GNRs, as shown in Fig. 4c. The widths of the GNRs changed depending upon the diameters of the Si NWs and the graphene RIE times, demonstrating that this method is highly controllable. Figure 4d shows AFM images of about 500 and 100 nm wide GNRs. Noting that NW assembly based on dielectrophoresis is a well-known tool for fabricating NW device arrays [30, 31], these results suggest that this technique can be easily adapted for fabricating single GNR arrays over large area.

**Fig. 5:**
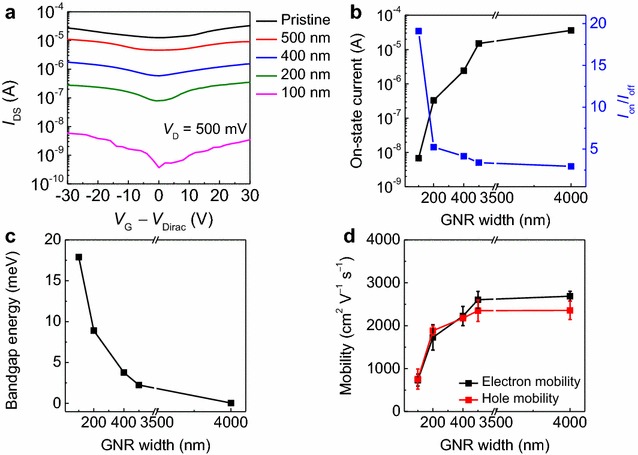
**a** *I*_DS_–*V*_G_ characteristics of GNR FETs with various GNR widths. **b** On-state current and on/off current ratio, and **c** bandgap energy of GNR FETs as a function of GNR width. **d** Electron and hole field-effect mobilities of GNR FETs as a function of GNR width

The electrical characteristics of GNR FETs with various widths were measured at room temperature. As shown in Fig. 5a, transfer characteristics of the devices strongly depend on the ribbon widths. Compared to wider GNR devices with similar channel lengths, a 100-nm wide GNR delivers much less current in the on state, but shows a significantly increased on/off current ratio (Fig. 5b). It is obvious from Fig. 5b that the on/off current ratio is inversely proportional to GNR width, e.g., the on/off current ratio for 500-nm wide GNR FETs is only 3.5 but gradually increases to 20 with decreasing ribbon width to 100 nm. This trend was consistent with previous reports on GNRs prepared using e-beam lithography and those chemically derived from carbon nanotubes [8, 9, 12]. The bandgap energy (*E*_g_) of GNRs has an exponential relationship with the on/off current ratio of GNR FETs (*I*_on_/*I*_off_  =  *C* exp(*E*_g_/*k*T)) [32, 33]. Theoretical calculations have suggested that the formation of GNRs opens up bandgaps that are inversely proportional to the ribbon widths (*E*_g_ = *α*/*W*), where *α* is between 0.3 and 1.5 [32, 33]. As shown in Fig. 5c, the bandgap energy increased with increasing on/off current ratio by narrowing the width of the GNRs. These results demonstrate that the opening of the bandgap in GNRs is due to the enhanced carrier confinement in the transverse direction and the edge effect when the GNR width is reduced. It is believed that the on/off current ratio can be further increased when NW masks with smaller diameter are used. The carrier mobility of the devices was estimated using the formula,

$$\mu _{{FE}} = \frac{{g_{m} L}}{{WCV_{{DS}} }}$$

where *g*_m_ is the transconductance, *L* is the channel length, *W* is the channel width, and *C* is the gate capacitance per unit channel length of a GNR. Since the fringe effect on capacitance at the ribbon edges of the GNRs must be considered due to the narrow ribbon widths of the GNRs, a simulation model based on finite element analysis reported previously was employed to obtain the capacitance [16, 34, 35]. As shown in Fig. 5d, the electron and hole mobilities decreased with decreasing ribbon width, approaching 730 and 760 cm^2^ V^−1^ s^−1^, respectively, at room temperature, due to the carrier scattering at GNR edges. These mobilities are comparable to those reported previously [36–38].

**Fig. 6:**
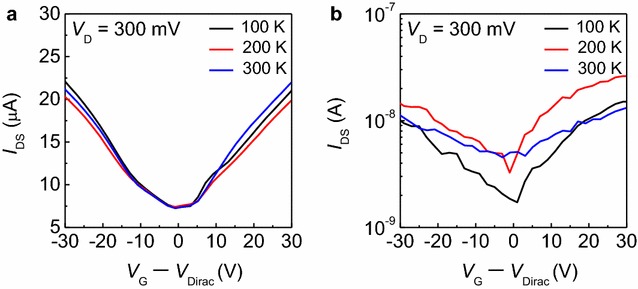
*I*_DS_–*V*_G_ characteristics of FETs based on **a** unpatterned graphene and **b** 100-nm wide GNR, measured at temperatures of 100–300 K

Figure 6 shows the temperature dependence of the transfer curves of pristine graphene and GNR FETs. In the case of pristine graphene FETs, the temperature dependence of the transfer curves was negligible because pristine graphene is semimetallic with a zero bandgap. However, as graphene was patterned into GNR, the off-state current was reduced due to suppression of a thermionic-emission current with decreasing temperature, leading to strong temperature dependence of the current. Therefore, the on/off current ratio increased with decreasing temperature since the charge carriers do not have sufficient energy to tunnel through the gap or to hop edge states at low temperatures, i.e., *k*T << *E*_g_, where *k* is the Boltzmann constant and *T* is absolute temperature. This confirms that structuring a graphene sheet into a ribbon can open the bandgap.

## Conclusions

In summary, GNRs with different widths were fabricated in a highly controllable manner, and their electrical transport properties were investigated. The GNRs were prepared from CVD-grown graphene sheets by using etch masks based on Si NWs synthesized from Si substrates by a combination of nanosphere lithography and metal-assisted chemical etching, and aligned between two electrodes by an electric-field-assisted alignment method, producing GNR FETs. The ribbon widths of the GNRs were controlled by changing the diameters of the Si NWs and the graphene etching times. The electrical transport characteristics of GNRs were found to be highly dependent on the GNR width: (1) the on/off current ratio was inversely proportional to GNR width; (2) the electrical properties of the GNR FETs showed a strong temperature dependence of the *I*_DS_–*V*_GS_ curves on ribbon width; (3) the field-effect mobility decreased with decreasing width of the GNR; and (4) the bandgap increased with increasing on/off current ratio by narrowing the width of the GNR. These results demonstrate the opening of the bandgap in graphene due to enhanced carrier confinement in the transverse direction and the edge effect in the ribbon structure.
